# Supplementary figures and images for: Loss of transcriptional heterogeneity in aged human muscle stem cells
Source: PLoS One. 2023 May 16;18(5):e0285018. doi: 10.1371/journal.pone.0285018 (PMC10187936; doi:10.1371/journal.pone.0285018)

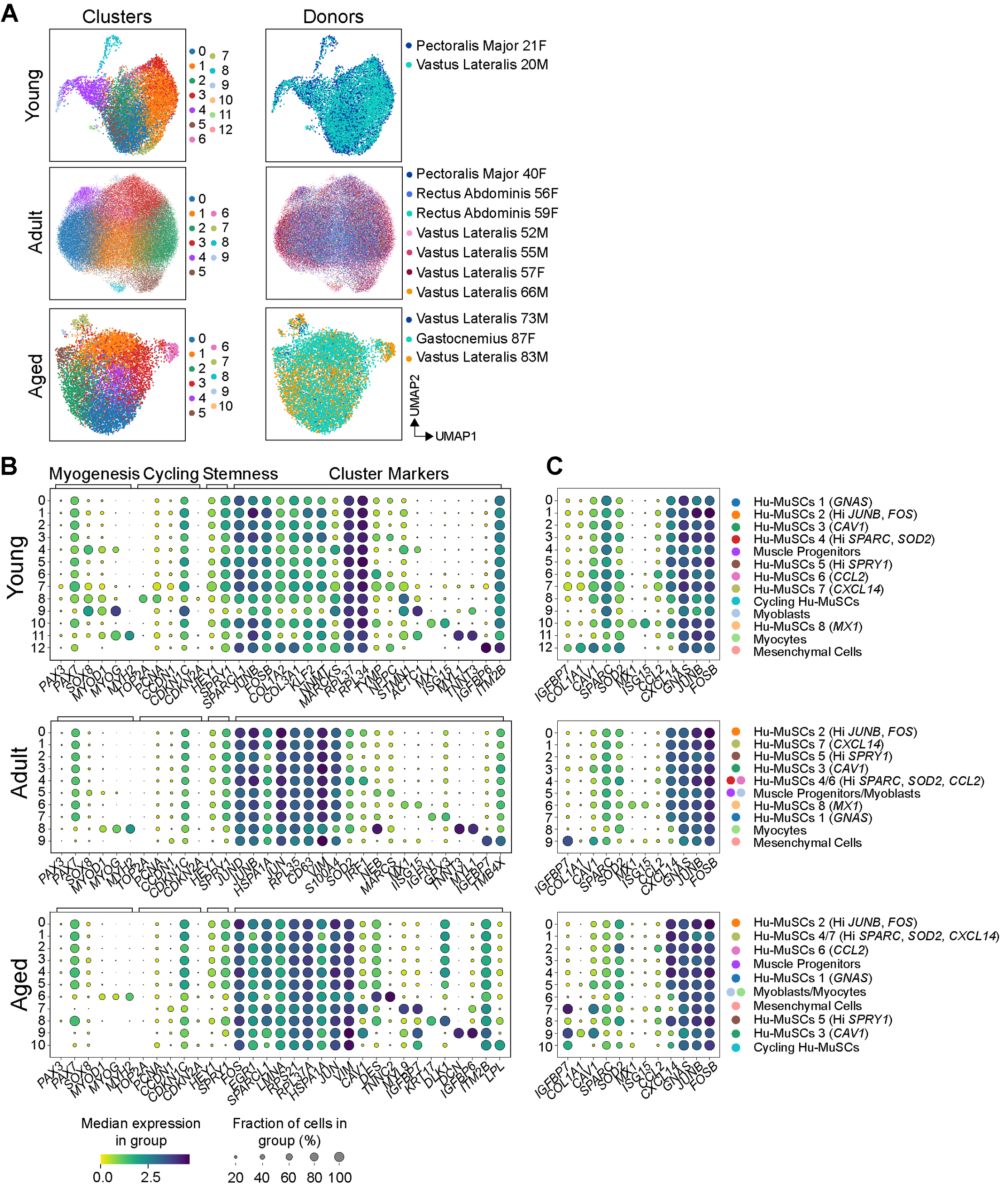

Supplement: S1 Fig — (A) As shown in Fig 1A, samples were first merged into their own age group. UMAP displaying clusters and samples for each age group. (B) Dot plots displaying the expression of myogenic, cycling, stemness and cluster marker genes for each cluster in each age group. (C) Dot plots displaying the expression levels of an identical gene set in the young, adult and aged Hu-MuSCs. (TIF) [file pone.0285018.s001.tif]

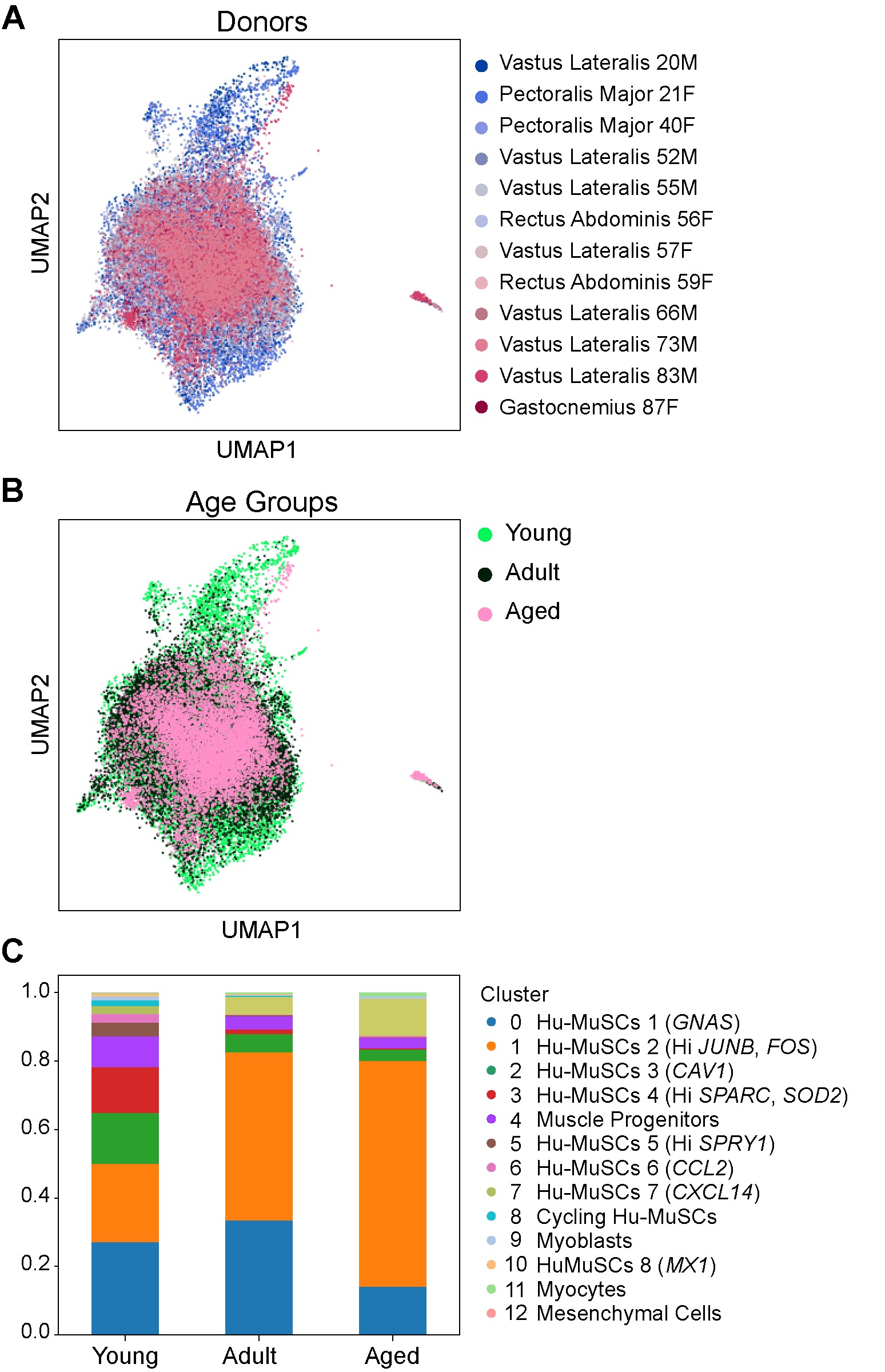

Supplement: S2 Fig — (A) UMAP of the INGEST analysis displaying all 12 samples. (B) MAP of each age group and their distribution in clusters. (C) Proportion plot of cells assigned to each cluster for each age group. (TIF) [file pone.0285018.s002.tif]

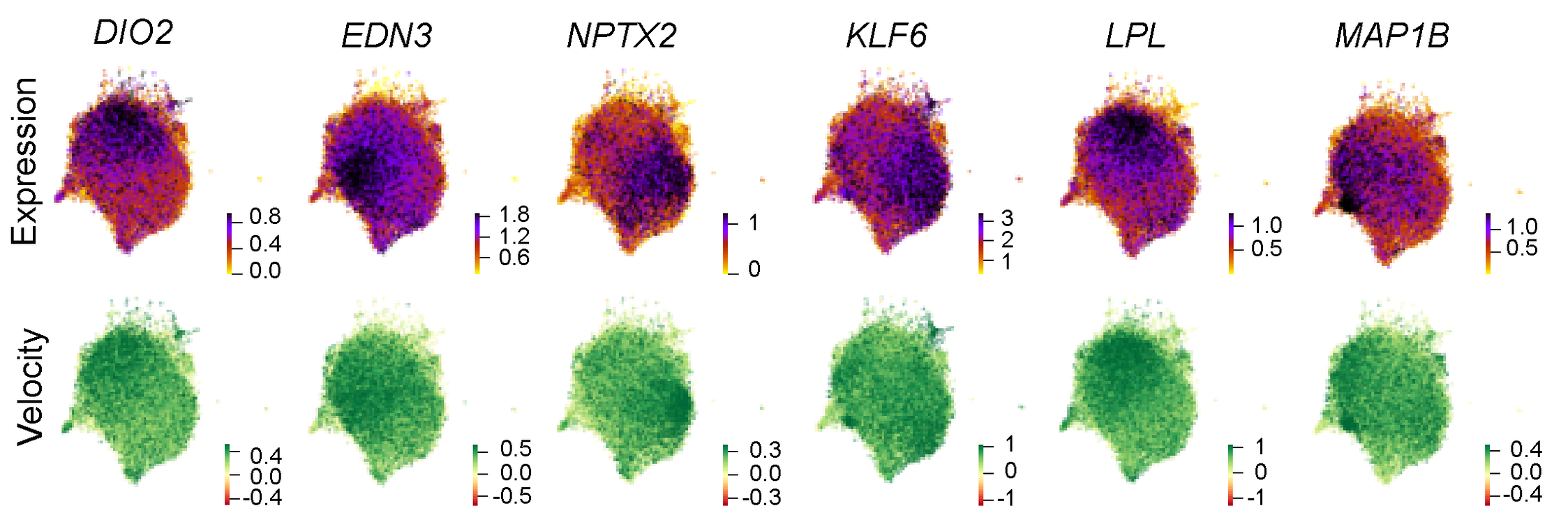

Supplement: S3 Fig — (TIF) [file pone.0285018.s003.tif]

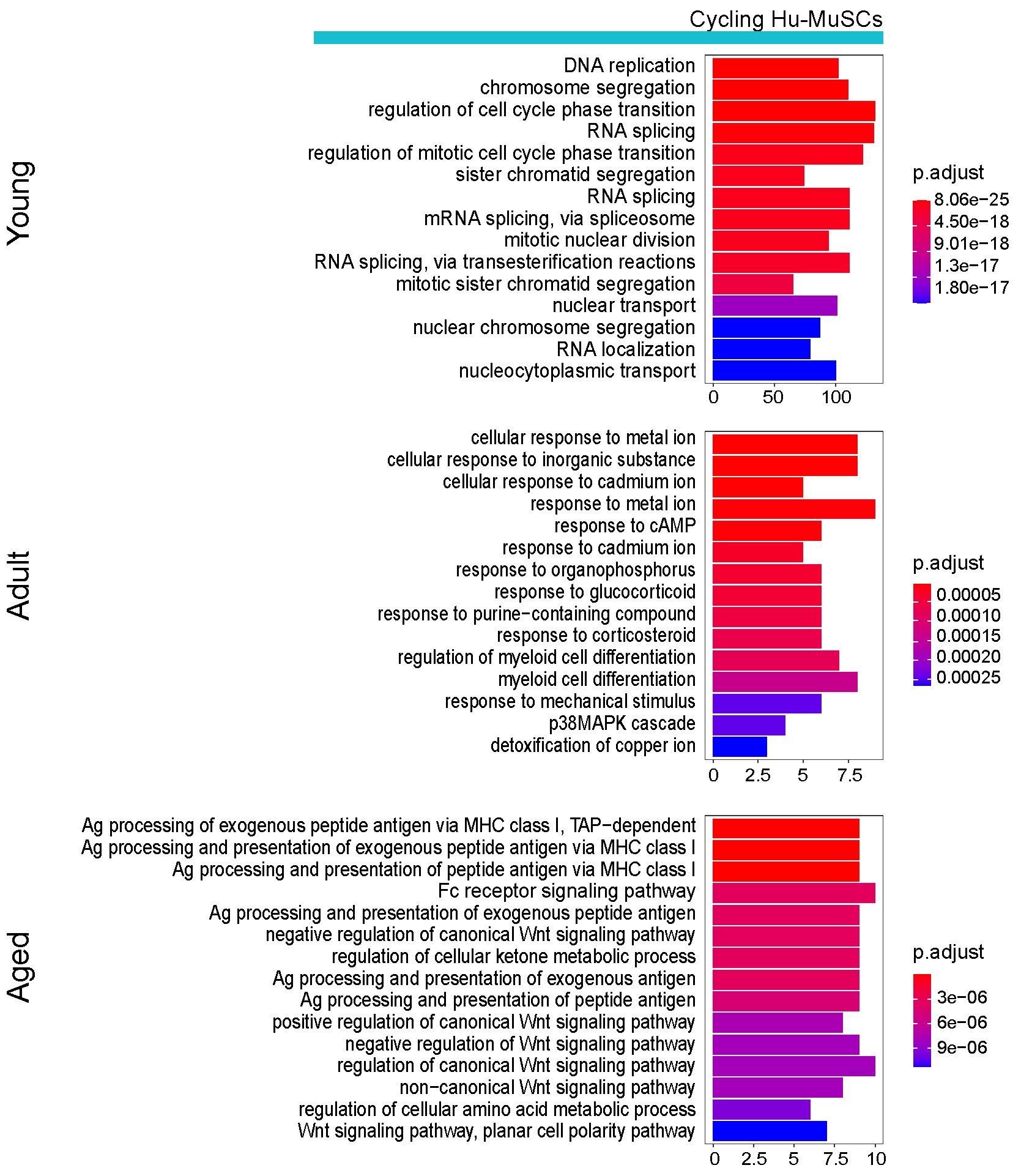

Supplement: S4 Fig — Bar plots of gene ontology analysis of differentially up-regulated genes in the cycling Hu-MuSCs cluster (8) for each age group. (TIF) [file pone.0285018.s004.tif]

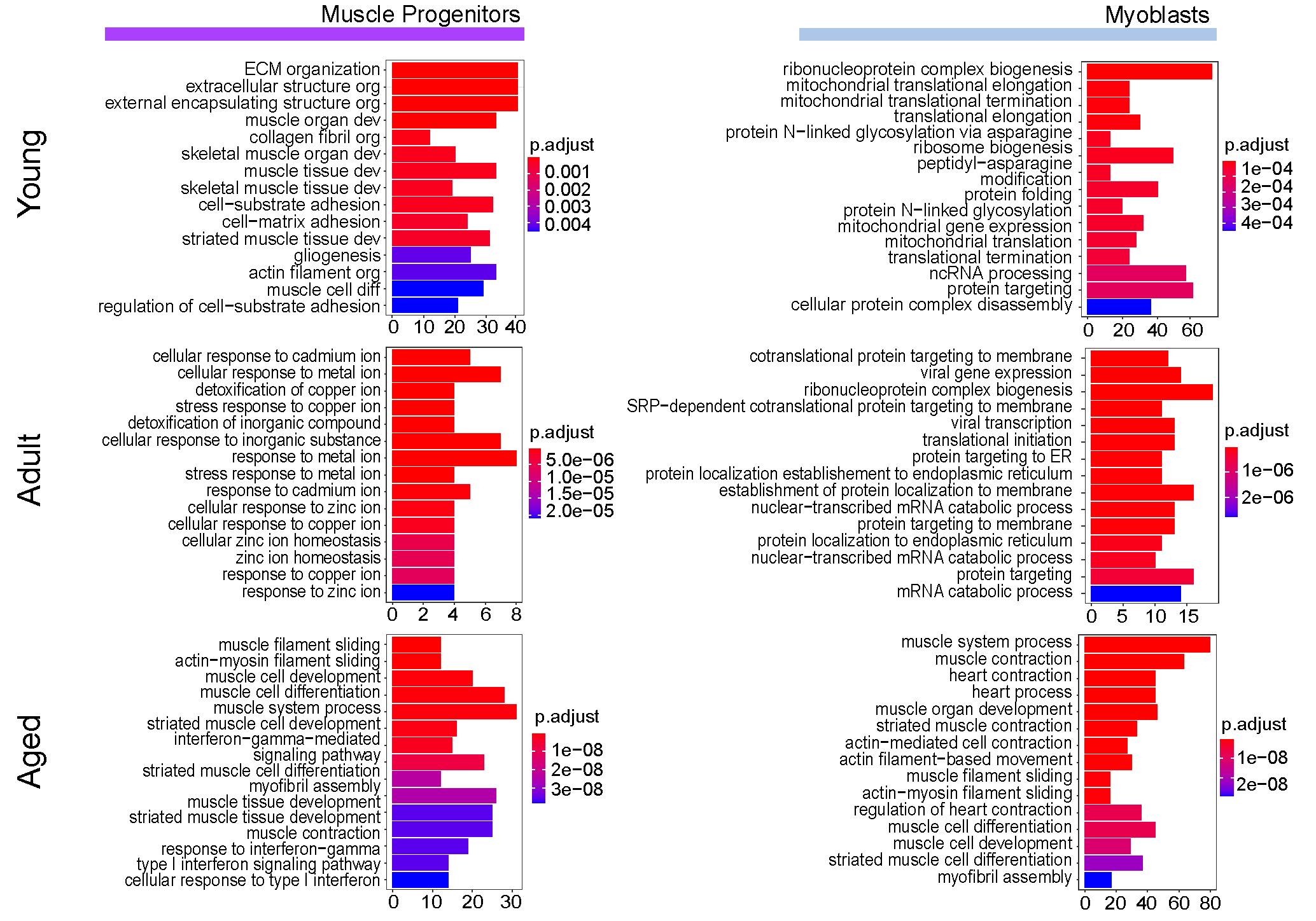

Supplement: S5 Fig — Bar plots of gene ontology analysis of differentially up-regulated genes in the muscle progenitor cluster (4), and myoblasts cluster (9) for each age group. (TIF) [file pone.0285018.s005.tif]

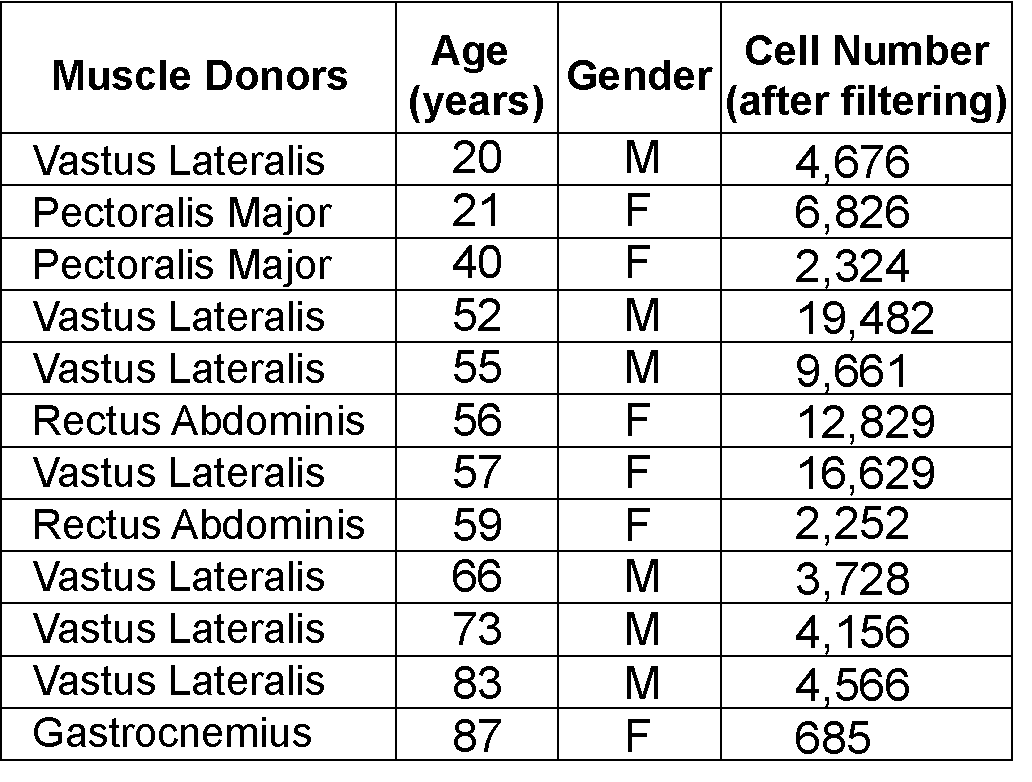

Supplement: S1 Table — (TIF) [file pone.0285018.s006.tif]
